# On the Cholesterol Raising Effect of Coffee Diterpenes Cafestol and 16-*O*-Methylcafestol: Interaction with Farnesoid X Receptor

**DOI:** 10.3390/ijms25116096

**Published:** 2024-05-31

**Authors:** Elena Guercia, Federico Berti, Rita De Zorzi, Luciano Navarini, Silvano Geremia, Barbara Medagli, Marco De Conto, Alberto Cassetta, Cristina Forzato

**Affiliations:** 1 Aromalab, illycaffè S.p.A., Area Science Park, Località Padriciano 99, 34149 Trieste, Italy; elena.guercia@illy.com (E.G.); luciano.navarini@illy.com (L.N.); 2 Department of Chemical and Pharmaceutical Sciences, University of Trieste, Via Giorgieri 1, 34127 Trieste, Italy; fberti@units.it (F.B.); sgeremia@units.it (S.G.); bmedagli@units.it (B.M.); marco.deconto@phd.units.it (M.D.C.); 3 CNR-Institute of Crystallography, Area Science Park, SS. 14, Km 163.5, Basovizza, 34149 Trieste, Italy; alberto.cassetta@cnr.it

**Keywords:** cafestol, 16-*O*-methylcafestol, coffee, FXR, fluorescence, circular dichroism

## Abstract

The diterpene cafestol represents the most potent cholesterol-elevating compound known in the human diet, being responsible for more than 80% of the effect of coffee on serum lipids, with a mechanism still not fully clarified. In the present study, the interaction of cafestol and 16-*O*-methylcafestol with the stabilized ligand-binding domain (LBD) of the Farnesoid X Receptor was evaluated by fluorescence and circular dichroism. Fluorescence quenching was observed with both cafestol and 16-*O*-methylcafestol due to an interaction occurring in the close environment of the tryptophan W454 residue of the protein, as confirmed by docking and molecular dynamics. A conformational change of the protein was also observed by circular dichroism, particularly for cafestol. These results provide evidence at the molecular level of the interactions of FXR with the coffee diterpenes, confirming that cafestol can act as an agonist of FXR, causing an enhancement of the cholesterol level in blood serum.

## 1. Introduction

Coffee is one of the most widely consumed beverages in the world, and in the years 2020/2021 it was estimated that around 166.63 million 60 kg bags of coffee were consumed worldwide [1].

Due to the large consumption, the physiological effects of the main compounds present in coffee have been extensively studied. Most of the studies regard caffeine [2], which is present in different amounts in the two main commercial coffee species, *Coffea arabica* (commonly known as Arabica) and *Coffea canephora* (commonly known as Robusta), with a concentration of 0.9–1.3% *w*/*w* in Arabica and 1.5–2.5% *w*/*w* in Robusta. Apart from caffeine, more than 1000 different compounds have been identified in coffee beans, and among them, chlorogenic acids (CGAs) and diterpenes received great attention due to their different physiological effects on human health [3,4,5].

The amount of chlorogenic acids varies depending on the coffee species. Robusta is richer, with an amount in the range of 6.1–11.3% *w*/*w*, while Arabica has a chlorogenic acid proportion in the range of 4.1–7.9% *w*/*w*. CGAs showed several beneficial effects on human health such as anti-diabetic, anti-carcinogenic, anti-inflammatory, and anti-obesity [6,7,8].

Diterpenes are present in the lipid fraction, also known as coffee oil, and their content in coffee beans is in the range of 0.5–1.2% *w*/*w* in Arabica and 0.2–0.8% *w*/*w* in Robusta [9].

**Figure 1:**
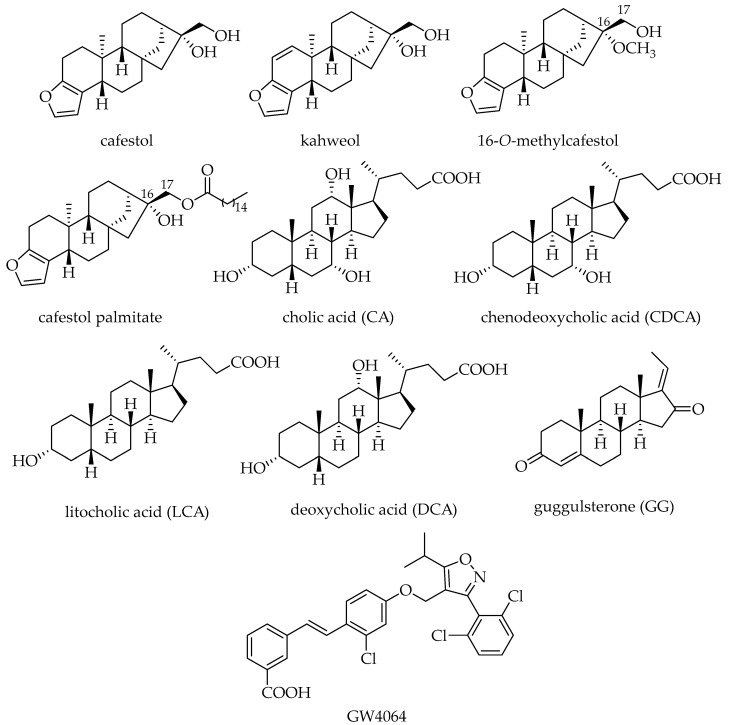
Structures of coffee diterpenes and of endogenous agonists CA, CDCA, LCA, and DCA, non-steroidal synthetic agonist GW4064, and antagonist GG of FXR.

Coffee diterpenes belong to the kaurene family [10], with cafestol (CAF), kahweol, and 16-*O*-methylcafestol (16OMC) being the most representative (Figure 1), although their presence is characteristic of the coffee species. In fact, *C. arabica* contains CAF and kahweol while *C. canephora* contains mainly CAF with kahweol and 16OMC present only in trace amounts. Since 2018, 16OMC has been considered a marker of *C. canephora* since it was never detected before in *C. arabica* [11]. These are mainly present in coffee as esters of palmitic acid or other fatty acids at the C17 position, while the free form is only in 0.4% *w*/*w* of the coffee lipid fraction [12]. They possess both positive and negative effects on human health, and considering the high consumption of the coffee brew, it is important to understand the impact on the human health of coffee diterpenes [13]. Anti-carcinogenic, anti-angiogenic, anti-inflammatory, anti-diabetic, and anti-oxidative stress effects were observed in subjects drinking a moderate amount of coffee or a brew with a moderate diterpene content. On the contrary, in heavy drinkers, or when drinking brews with a high amount of diterpenes, like Scandinavian boiled coffee, negative effects were recorded. Among these are an increased concentration of lipids or cholesterol in serum, an amplified risk of cardiovascular diseases such as aortic valve stenosis, and the development of thrombosis [14,15,16].

The content of diterpenes in coffee does not depend only on the coffee species. Several other factors influence the presence of these compounds in the final beverage, such as the geographical origin, ripeness of the beans, agricultural practices, roasting, and even beverage preparation. In particular, diterpenes are partially retained by paper or popular filters [17], while they are present in larger amounts in unfiltered coffee brews such as Scandinavian boiled coffee, the cafetière coffee (French-press), and Turkish coffee (3–6 mg of each diterpene per cup), and all the preparations that do not employ paper [18].

Thelle et al. observed the correlation between coffee consumption and cholesterol rise for the first time in 1983 [19], with an increase that depends on the brewing method. In particular, CAF represents the most potent cholesterol-elevating compound known in the human diet, being responsible for more than 80% of the effect of coffee on serum lipids [20]. Further studies demonstrated that a dose of 10 mg/die of CAF for 4 weeks increases serum cholesterol by 0.13 mmol/L, an 8–10% increase [21]. In 2022, the association between serum total cholesterol (S-TC) and espresso coffee consumption was confirmed to be significantly stronger for men than for women. Boiled/plunger coffee was associated with a similar increase of S-TC in both sexes. Filtered coffee was associated with a small increase in S-TC in women [22]. The hyperlipidemic effect is linear with the cafestol dose and several hypotheses have been proposed for the mechanisms of action of this diterpene on lipoproteins: (1) a direct effect of CAF on the sterol regulatory element binding proteins (SREBP)-pathway [23,24,25,26,27]; (2) increased activity of cholesterol ester transfer proteins (CEPT) and phospholipid transfer protein (PLTP), and a simultaneous decrease of the activity of the lecithin:cholesterol acyltransferase [28]; (3) an antagonistic effect on peroxisome proliferator-activated receptors (PPARs) [29]. The most plausible mechanism of cafestol-induced cholesterol increase in humans is via the SREB pathway. However, data from different studies are inconsistent and suggest that CAF or its metabolites may act differently in various cell types. In 2007, Ricketts et al. showed that CAF is an agonist of Farnesoid X Receptor (FXR) and specifically activates FXR and Pregnane X Receptor (PXR), both in vivo in a mice model system and in vitro in a cell model, thus suppressing the expression of key genes involved in cholesterol homeostasis [30].

FXR belongs to the superfamily of nuclear receptors, activated upon binding with specific ligands that regulate several important aspects of mammalian physiology. Two FXR protein families have been identified in animals, namely FXRα and FXRβ. Members of the former are evolutionarily conserved among species (from fish to humans) and are highly expressed in the liver, intestine, kidney, and adrenal glands, with low levels of expression in the adipose tissue and heart [31]. FXR has different roles in humans, from the regulation of bile acid homeostasis (FXR inhibits bile acid synthesis in the liver) to an effect on lipid, glucose, and amino acid metabolism [31,32]. Indeed, if dietary cholesterol reaches elevated intracellular levels, the activation of FXR regulates the transcriptional programs of the cell through a negative feedback mechanism, resulting in the reduction of bile acid toxicity in the liver, intestine, kidneys, and adrenal glands [33]. In its inactive form, the receptor is bound to specific DNA sequences, namely the FXR responsive elements (FXRE), in a heterodimeric form with its co-receptor Retinol X Receptor (RXR). When activated, FXR releases the DNA sequence, thereby reducing the intracellular bile acid content through four different mechanisms. (1) FXR activates the expression of the Small Heterodimer Partner (SHP), which in turn represses the expression of a member of the Cytochrome P450 family (CYP7A1) responsible for the rate-determining step of the conversion of cholesterol in bile acids [34]. (2) Agonist-bound FXR upregulates the expression of FGF19, a suppressor of CYP7A1 [35]. (3) FXR contributes to the reduction of the intracellular content of bile acids by activating the expression of their efflux pump BSEP in the hepatocyte canalicular membrane [36] while at the same time (4) inhibiting the expression in the intestine of ASBT, the apical sodium-dependent bile acid cotransporter, responsible for the cellular uptake of bile acids, through a more complex mechanism involving SHR [37].

Structurally, FXR is similar to most nuclear receptors, with an N-terminal domain, a DNA binding domain, and a ligand-binding domain (LBD), the latter composed of 11 α-helices and 4 β-strands forming a hydrophobic binding pocket. Its endogenous ligands are conjugated and unconjugated bile acids such as cholic acid (CA) and chenodeoxycholic acid (CDCA), synthesized in the liver, and their derivatives lithocholic acid (LCA) and deoxycholic acid (DCA) (Figure 1). The potency of bile acids in activating FXR is ranked as CDCA > DCA> LCA > CA [38].

To improve our understanding at the molecular level of the correlation between CAF intake and cholesterol increase, the present study focuses on the investigation of CAF and 16OMC binding to FXR through fluorescence spectroscopy and circular dichroism analysis. Experimental results are rationalized by comparison with docking and molecular dynamics simulations.

## 2. Results

### 2.1. Wild-Type FXR-LBD and Stabilizing Mutations

**Figure 2:**
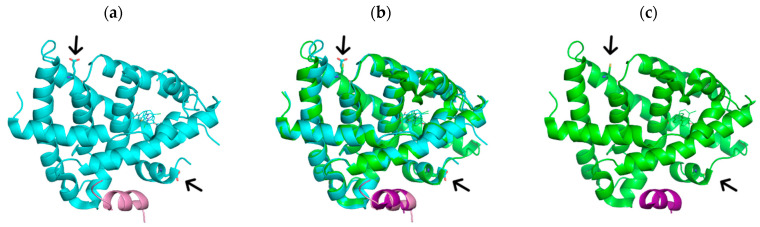
Comparison between the crystal structures of the mutated C432E/C466E LBD of FXR (**a**), from the structure with PDB code 6A5Z (in cyan), and the wild-type LBD (**c**) from the structure with PDB code 6A5Z (in green). Panel (**b**) shows the superimposition between the two structures. Arrows highlight the positions of the mutated residues. The helical structures in magenta and pink represent the binding peptide used in crystallization experiments.

The LBD of FXR, corresponding to residues 225 to 472, was expressed in *E. coli* cells as a recombinant protein with an N-terminal His tag. Initial binding tests using this protein, at high concentration, with diterpenes revealed a low stability in solution of the complexes. Occurrence of precipitation was reduced upon addition of reducing agents such as dithiothreitol or β-mercaptoethanol. Therefore, a stabilized mutant was produced by introducing two mutations reported in the published structures (PDB structures 3HC6, 3P89, 4OIV, 3GD2, 6A5Z, and 6A60, among others): superficial cysteine residues 432 and 466, prone to oxidation, were mutated to glutamate through sequential site-directed mutagenesis (Figure 2a, from the crystal structure with PDB code 6A5Z), obtaining the construct named CCEE.

### 2.2. Fluorescence Measurements

**Figure A1:**
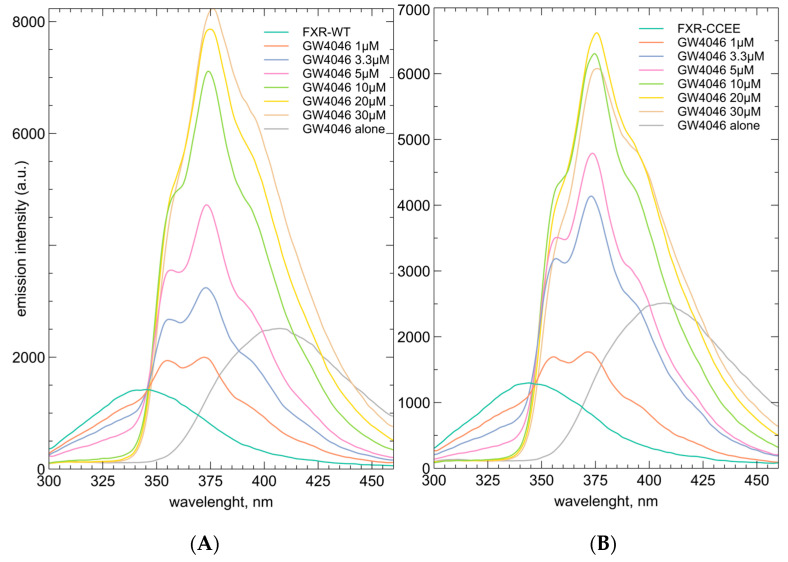
Emission (λ_exc_ = 280 nm) spectra of 10 μM solutions of FXT-WT (**A**) and FXR-CCEE (**B**) in phosphate buffer, in the absence of ligands and in the presence of increasing concentrations of GW4046 ranging from 1 μM to 30 μM.

**Figure A2:**
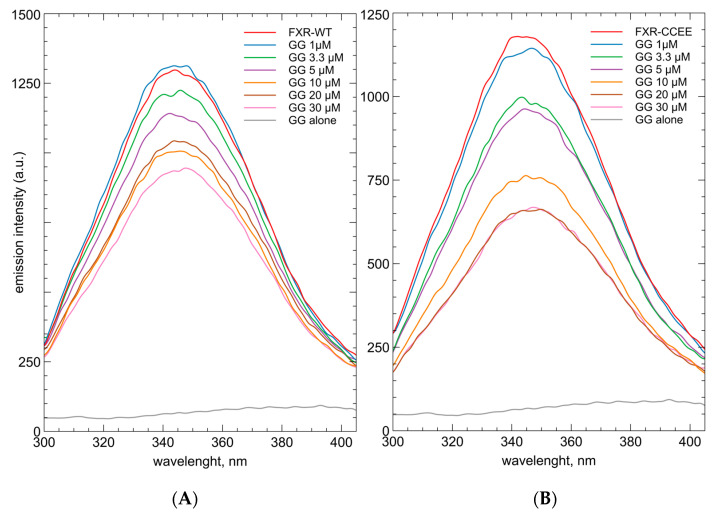
Emission (λ_exc_ = 280 nm) spectra of 10 μM solutions of FXT-WT (**A**) and FXR-CCEE (**B**) in phosphate buffer, in the absence of ligands and in the presence of increasing concentrations of GG ranging from 1 μM to 30 μM.

The change of intrinsic fluorescence emissions of the proteins in the presence of ligands was measured to compare the behavior of the in-house expressed protein with the literature on the reference agonists of FXR as GW4064 and GG and to study the interactions of CAF and 16OMC. We replicated the titrations reported by Yang et al. with both FXR-WT and FXR-CCEE ligand-binding domains [39]. The results on the FXR-WT were almost identical to the literature, with a very unusual red shift and a large increase of emission upon interaction with GW4064, and a decrease without shifts in the maximum emission wavelength with GG (See Appendix A, Figure A1 and Figure A2). The behavior of the FXR-CCEE was very similar to the FXR-WT, with close quenching parameters in all cases, being the Stern–Volmer constants for GW4064 equal to 788 × 10^3^ ± 20 × 10^3^ and 757 × 10^3^ ± 35 × 10^3^ L·mol^−1^ for the FXR-WT and FXR-CCEE proteins respectively. With GG, the numbers for the FXR-WT and the FXR-CCEE mutant were quite different, namely 33.65 × 10^3^ ± 5 × 10^3^ and 54.36 × 10^3^ ± 5 × 10^3^ L·mol^−1^, respectively.

**Figure 3:**
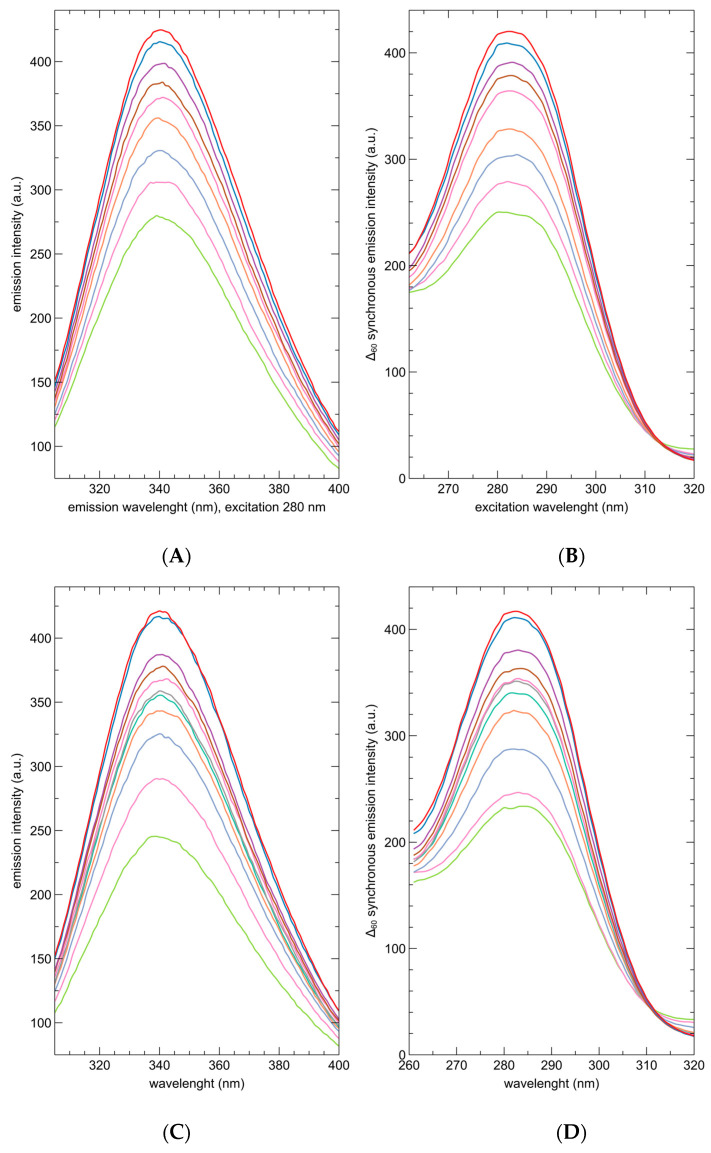
Emission (λ_exc_ = 280 nm) and synchronous spectra (Δ = 60 nm) of 1 μM solutions of the FXR-CCEE in phosphate buffer, in the absence of ligands (red spectra), and in the presence of increasing concentrations of CAF and 16OMC ranging from 100 nM (blue), 300 nM (purple), 500 nM (brown), 1 μM (pink), 3 μM (turquoise), 5 μM (ocher), 10 μM (light blue), 20 μM (magenta), 40 μM (green); evidence a decrease of the fluorescence signal upon binding of the diterpenes that is mainly related to the quenching of the tryptophan signal; (**A**): emission, CAF; (**B**): synchronous, CAF; (**C**): emission, 16OMC; (**D**): synchronous, 16OMC.

In addition, we studied the interactions of CAF and 16OMC with the FXR-CCEE protein. Fluorescence titrations are reported in Figure 3. The emission of the protein is largely decreased upon the addition of both ligands, without any shift in the maximum emission wavelength.

A comparison between the emission and the synchronous spectra shows that most of the quenching is related to the fluorescence of tryptophan residues rather than to that of tyrosine. This is consistent with an interaction occurring in the close environment of one or both tryptophan residues of the protein (W454 and W459), located on the helices 11 and 12 at the bottom of the binding site.

**Figure 4:**
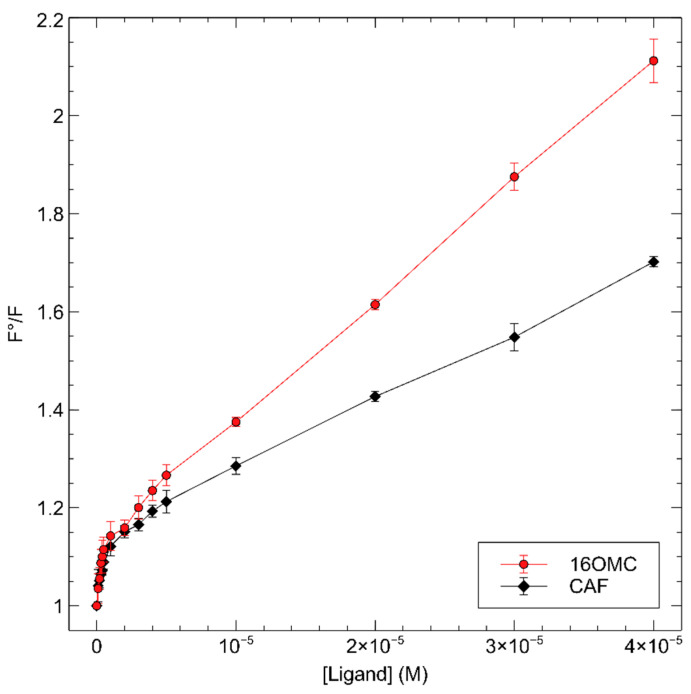
Stern–Volmer plots for the fluorescence emission quenching of 1 μM FXR-CCEE upon titration with CAF and 16OMC highlight a bimodal behavior at low and high concentrations of the ligand.

The quenching data were submitted to Stern–Volmer analysis (Figure 4).

**Figure A3:**
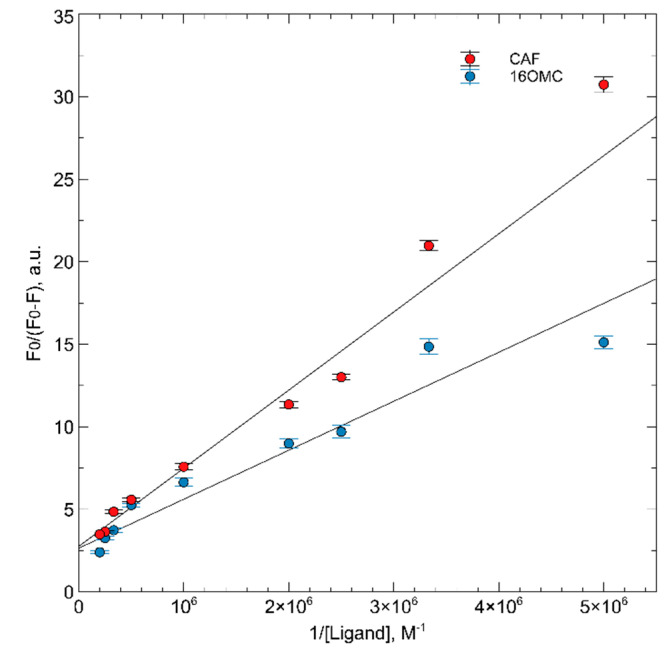
Lehrer plot for the fluorescence quenching of intrinsic emission of FXR-CCEE with CAF and 16OMC.

The plots highlight a bimodal behavior of the quenching phenomenon, with two linear regions (Figure 4 and Appendix A, Figure A3). The first occurs at low concentrations of ligands, with a higher slope, and the second at higher concentrations, with a lower slope. The change of slope occurs at about 1 μM in both cases.

We analyzed the two regions separately, and the data are reported in Table 1.

The quenching constants are consistent with a static mechanism in both the ranges and for both the terpenes, while the contributions from dynamic quenching seem negligible, as in all cases, the values are well above the threshold value of 10^10^ L mol^−1^ s^−1^.

### 2.3. Molecular Docking and Molecular Dynamics Analysis of the Positions of the Trp Residues

**Figure 5:**
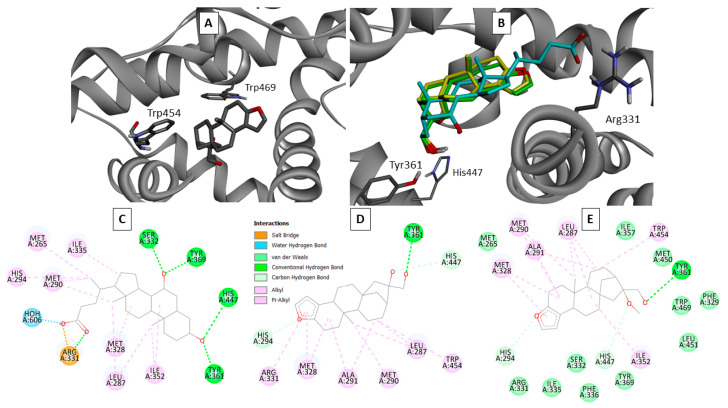

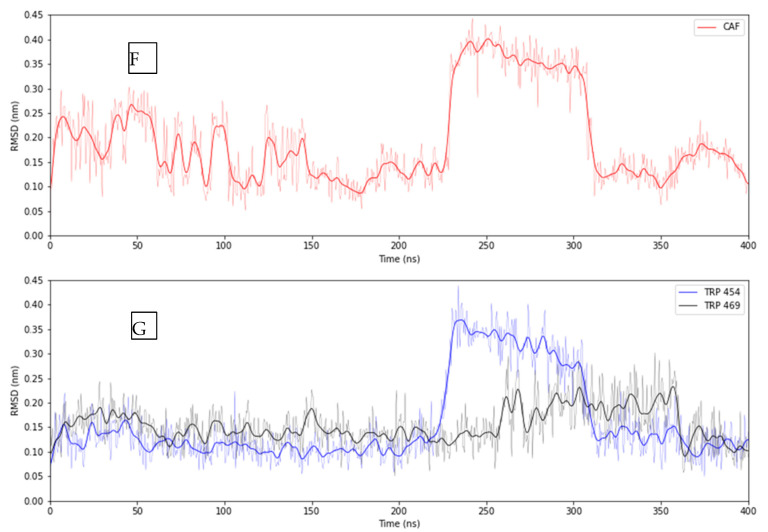
Docking analyses of CAF and 16OMC inside the binding site of FXR support the hypothesis that the ligand sits in close proximity to Trp 454 but not to Trp 469. MD simulation results confirm the mobility of the former residue. (**A**): best docking pose for CAF after optimization (Oxygen atoms are colored in red, nitrogen in blue, and hydrogen in white). (**B**): overlay of the best docking poses for CAF (green), 16OMC (yellow), and of CDCA (blue) inside FXR. (**C**–**E**): interaction maps for CDCA (**C**), CAF (**D**), and 16OMC (**E**). (**F**): 400 ns MD simulation of the CAF ligand bound to the LBD of FXR, RMSD of the CAF molecule along the trajectory. (**G**): RMSD of the Trp residues along the trajectory. Thick lines represent the curves smoothed using a Gaussian filter at 3.0 σ.

We have carried out docking of CAF and 16OMC inside the binding site of FXR, starting from the crystallographic structure of the complex of CDCA and FXR (pdb id. 6HL1), and the resulting models are reported in Figure 5.

Both the terpene ligands are found to partially occupy the binding site for CDCA (Figure 5B), placing their primary hydroxyl groups as that on the A ring of CDCA, which is at a hydrogen bond distance from Tyr 361 and His 447. Their aliphatic ring systems lie in the same space occupied by that of CDCA, establishing similar hydrophobic interactions (Figure 5C–E). However, CDCA also makes strong polar interactions at its carboxyl end, which are obviously impossible for the terpenes. Nevertheless, at the bottom of the site, the terpenes are close to the tryptophan side chains, mostly to Trp 454, and establish π–alkyl interactions with its indole system. Conversely, Trp 469 is less close to the ligands, as it is located on the other side of Trp 454 (Figure 5A).

To further elucidate the interaction of the terpenes, we analyzed the behavior of the protein complexes with CDCA and with CAF in 400 ns molecular dynamics simulations. Calculations were performed starting from the models of the LBD of FXR with CDCA or CAF obtained from the docking analysis. The MD simulation of CDCA in the binding site of FXR-LBD did not show significant changes for either the position of the ligand or the positions of the Trp residues, as demonstrated by the RMDS of both the CDCA and the Trp residues along the trajectory of the simulation (See Appendix C, Figure A5, Movie S1). In this case, the ligand fully occupies the binding site and displays only minimal positional changes. On the contrary, in the simulation of CAF, two main positions of the ligand within the binding site of the protein can be distinguished (Figure 5), resulting from the mismatch between the binding pocket dimensions and the smaller ligand. Analyzing the changes of the Trp residues along the simulation, a clear correlation can be observed between the positional changes of the ligand and the shift of Trp 454 (see Appendix C, Movie S2), confirming the interaction between these two groups as predicted by the docking analysis. Figure 5F shows the RMSD of the cafestol ligand, and Figure 5G shows that of the two Trp residues.

### 2.4. Circular Dichroism Analysis

**Figure 6:**
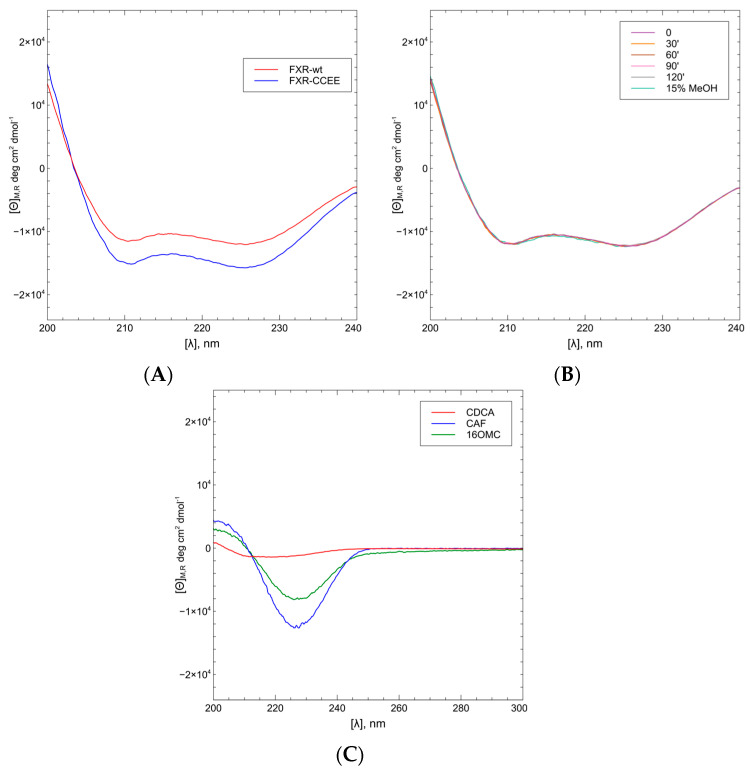
(**A**): CD spectra of WT-FXR (5 μM) and FXR-CCEE (5 μM) in PBS show a similar secondary structure content for the two forms; (**B**): CD spectra of FXR-CCEE in the time range 0–2 h and with 15% of methanol show that the protein retains the conformation and does not undergo denaturation in the presence of the alcohol; (**C**): CD spectra of CDCA, CAF and 16OMC in methanol are used as reference in the binding analysis.

The interaction between FXR-CCEE and the ligands CDCA, CAF, and 16OMC was determined by means of CD analysis in different conditions. At the beginning, the FXR-WT was used, and the CD spectra of the wild-type protein were registered upon subsequent addition of the ligand to a 5 μM solution of the protein in different buffers. Although several buffers were considered (Na_2_SO_4_/phosphate; PBS (no Gly); Tris buffer + Gly; DDT/buffer; buffer beta-mercapto; TCEP in Na_2_SO_4_/phosphate), it was evidenced that the stability of the FXR-WT was not good upon addition of the ligand and a precipitate was observed. Further experiments were performed with the FXR-CCEE, resulting in very similar CD spectra compared to the wild-type (Figure 6A).

The stability of FXR-CCEE was verified over a time range of 0–2 h at 25 °C, with a higher percentage of methanol used in titrations with ligands, in order to verify that the solvent does not affect the conformation of the protein. As can be seen from Figure 6B, no changes in the conformation of the protein over time or in the presence of 15% methanol were observed since all CD spectra perfectly overlapped.

The CD spectra of FXR-CCEE (5 μM) upon addition of CAF and 16OMC, with protein:ligand ratios in the range 10–100 (ligand concentrations 50 μM, 250 μM, and 500 μM), were compared with the CD spectra of FXR-CCEE upon addition of CDCA in the same range of the protein/ligand ratios. To determine the CD spectra of only FXR-CCEE, it is necessary to also consider the contribution of the ligands in the analyzed region since they present a Cotton effect in the same range (Figure 6C). For this purpose, all spectra were subtracted with the corresponding blank, which consisted of the buffer solution containing the ligand in methanol at the same final concentration. As can be seen from Figure 6C, both CDCA and diterpenes present a CD band in the range of 240–200 nm, although CDCA to a lesser extent with respect to the two diterpenes CAF and 16OMC.

**Figure 7:**
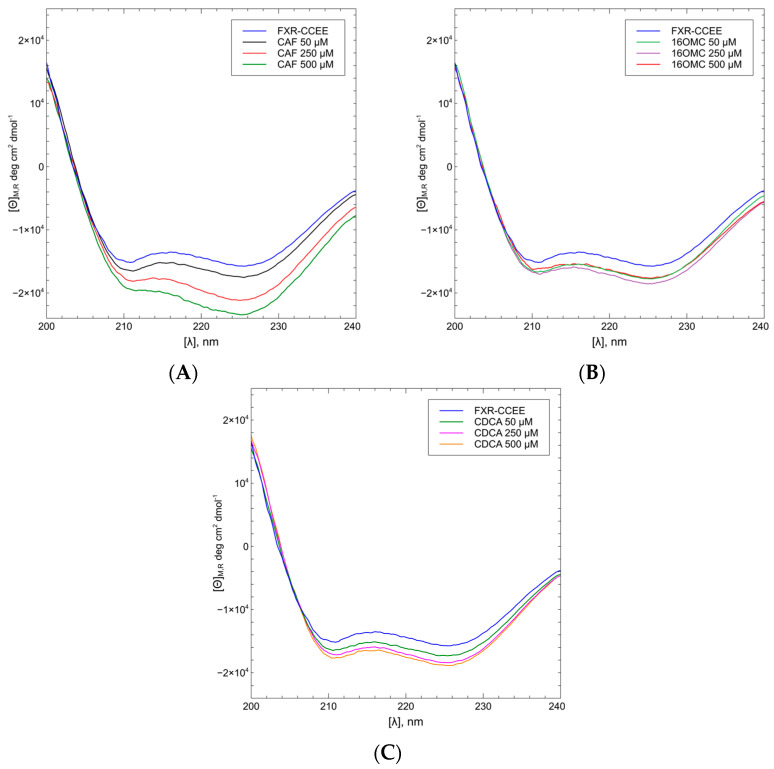
CD spectra of FXR-CCEE with the ligand CAF (**A**) show an increase in secondary structure content upon increasing the concentration of the terpene. Binding of 16OMC (**B**) or CDCA (**C**) determine only a minor change in the CD spectra of the protein.

CDCA, the natural agonist of FXR, showed a slight change in the protein conformation giving a higher intensity of the negative CD bands of the proteins upon increasing the concentration of the ligand. A similar conformational change was also observed with 16OMC, while CAF showed a remarkable conformational change, increasing the α-helix percentage from 59% to 68% (Figure 7).

To confirm the interaction between FXR-CCEE and the three ligands analyzed, the thermal unfolding of FXR-CCEE was analyzed.

**Figure 8:**
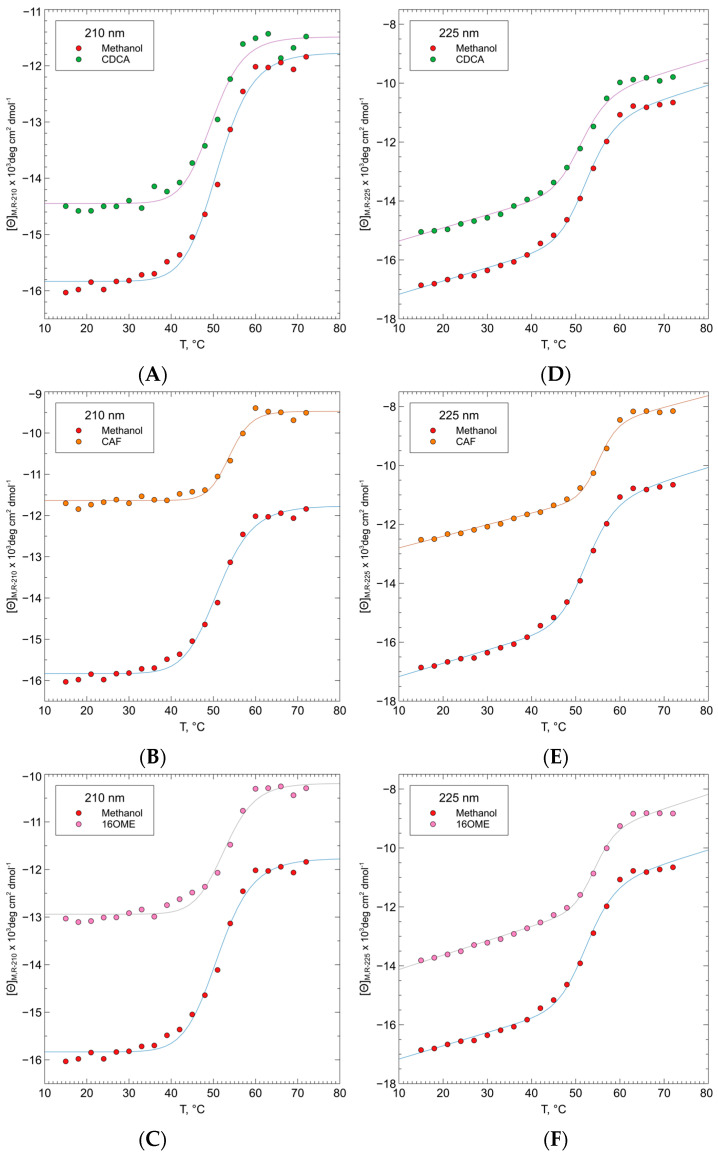
Thermal denaturation of FXR-CCEE with 15% methanol, as measured by CD at 210.2 and 225 nm and in the presence of CDCA (**A**,**D**), CAF (**B**,**E**), and 16OMC (**C**,**F**) is used to assess the stabilizing effect of the ligands on the protein folding.

The unfolding temperature of the FXR-CCEE was determined by the change of molar ellipticity at 201.2 and at 225 nm upon temperature in the range 15–72 °C, using FXR-CCEE with 15% of methanol at 5 μM and diterpenes at 40 μM, while CDCA was 50 μM. (Figure 8, Table 2).

### 2.5. Isothermal Titration Calorimetry (ITC) of FXR-CCEE

**Figure A4:**
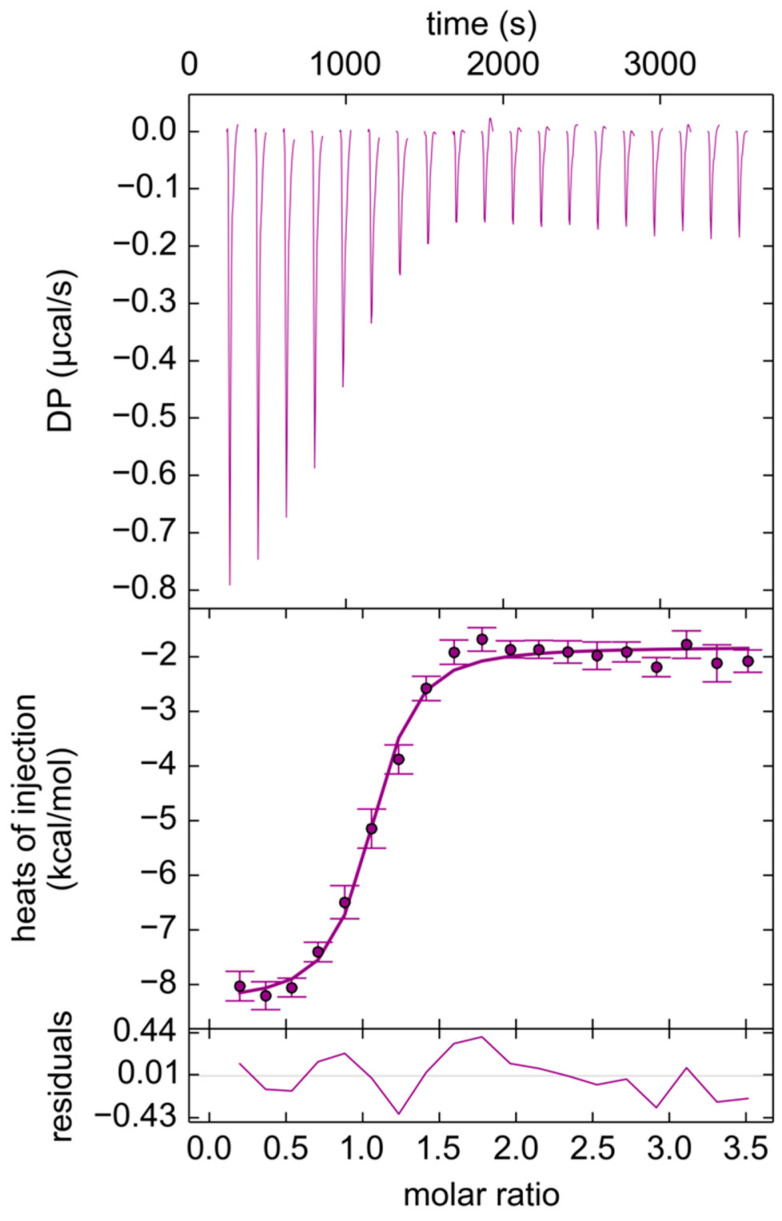
Thermogram (**upper panel**) and Titration Isotherm (**lower panel**) of GW4064 into FXR-CCEE solution.

**Figure A5:**
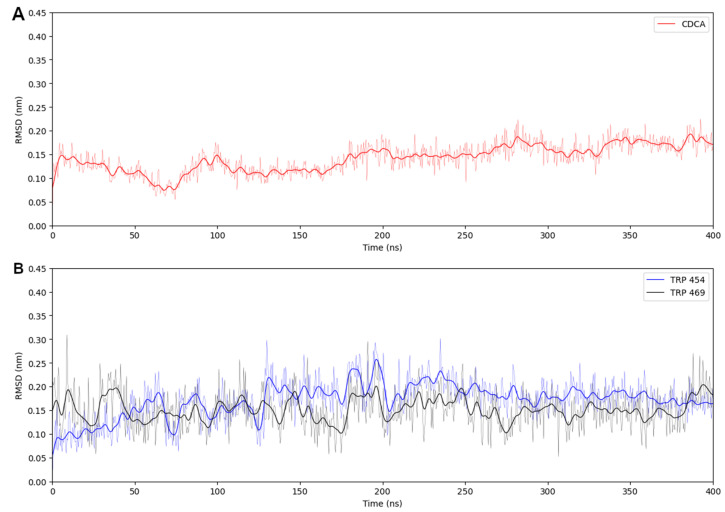
Four hundred nanosecond MD simulation of the CDCA ligand bound to the LBD of FXR. (**A**) RMSD of the CDCA molecule along the trajectory. (**B**) RMSD of the Trp residues along the trajectory, Trp 454 in blue and Trp 469 in grey. Thick lines represent the curves smoothed using a Gaussian filter at 3.0 σ.

Isothermal titration calorimetry was employed to further characterize the binding of cafestol to the ligand-binding domain of FXR. Results of the ITC measurements show that, while a significant exothermic binding is observed for GW4064 (see Appendix B, Figure A4, Table A1), no significant heat exchange can be measured when the protein is titrated with either CAF or the natural agonist CDCA.

## 3. Discussion

The wild-type LBD of FXR was expressed as a recombinant protein in *E. coli* cells. However, the low stability of this protein prompted us to introduce two stabilizing mutations from the literature, namely the mutation of two cysteine residues to glutamate. The comparison between the structure of the wild-type LBD (PDB code 3DCU, Figure 2b,c) and the mutated form CCEE reveals that the binding domain of the receptor is not affected by these stabilizing mutations, while the experimental behavior of the protein revealed an increased stability upon ligand binding.

To confirm the similar behavior of the mutant to the wild-type, we performed fluorescence measurements in the presence of the ligands GG and GW4064 and compared these results with the literature. While indicating an unusual behavior of the protein with the latter ligand, fluorescence measurements were consistent with the literature. Considering that the Stern–Volmer constants represent the equilibrium constants for the formation of the protein–ligand complexes, the mutant protein shows a better affinity for GG than the wild-type. We believe that the data on GG are more reliable than those on GW4064, as in our opinion, the unusual behavior of the fluorescence emissions observed with this ligand (the emission of the protein is apparently red-shifted from the usual 345 nm to 375 nm in the presence of the ligand) may be due to a combination of effects that cannot be treated with the usual Stern–Volmer analysis. The better affinity shown by the mutant protein for GG may be due to its better stability in solution.

The binding of the protein to the coffee diterpenes was studied through fluorescence spectroscopy, circular dichroism, and isothermal titration calorimetry. We used docking and molecular dynamics to interpret the experimental results at the molecular level.

In the binding experiments with CAF and 16OMC, Stern–Volmer plots for the fluorescence emission quenching (Figure 4) show a bimodal behavior closely similar to that observed by Yang et al., with GG as the quenching ligand [40]. In order to explain this phenomenon, they suggested that binding occurs in a way that allows fully close interactions with a fraction of tryptophan residues while the other fraction remains partly shielded. They analyzed the quenching data according to the Lehrer Equation (1):

(1) $$\frac{F_{0}}{F_{0}-F}=\frac{1}{\left[Ligand\right]\cdot K_{SV}\cdot f_{a}}+\frac{1}{f_{a}}$$

where *f_a_* is the fraction of fluorophore molecules available to the quencher without any shielding. We analyzed the data in the same way and obtained fa values of 0.44 ± 0.4 for CAF and 0.53 ± 0.5 for 16OMC. As the protein contains two tryptophan residues, this suggests that, in our case, just one of them is in close contact with the ligands in the binding site, as proposed by Yang for GG. In this hypothesis, the dissociation constant for the protein–ligand complexes should be more correctly given by the reciprocal of the low-range K_SV_ values and would result in the values 8.54 μM for CAF and 7.62 μM for 16OMC. Such values are comparable with those reported by Yang for prenylflavonoids [40] and for bile acids as well. However, in our opinion, another interpretation of the quenching data is possible for our ligands and GG: the change of slope occurs actually at ligand concentrations corresponding to a 1:1 molar ratio with the protein, and this could also be seen as a consequence of a tight-binding titration of a high-affinity binding site placed close to one tryptophan side chain. In this hypothesis, the affinity of CAF, 16OMC, and GG would be far more favorable, and a second binding site would be occupied in the high concentration range, with affinities in the 10 μM range.

Molecular docking (Figure 5) is consistent with the hypothesis that the ligand is in the vicinity of Trp 454 in the binding site. Molecular dynamics simulations suggest a significant change in the environment around this residue upon binding of the terpenes (Figure 5, Appendix C, Movie S2). On the contrary, the position of Trp 469 is not significantly influenced by the changes occurring during cafestol binding, confirming the interpretation of the fluorescence results.

Conformational changes related to ligand binding were observed by circular dichroism. 16OMC causes a slight conformational change of the protein, dependent on ligand concentration (Figure 7B), similar to the FXR natural agonist CDCA (Figure 7C). On the contrary, the binding of CAF increases the helix secondary structure content of the protein (Figure 7A).

Considering the enhancement in the α-helix content upon binding to CAF and to 16OMC to a minor extent, CD analyses were performed at different temperatures to assess whether the increase of backbone interactions of the helical secondary structure has a stabilizing effect on the protein. CD thermal unfolding analyses show that CAF and 16OMC determine a slight shift of the unfolding temperature to higher values (Figure 8), indicating that the ligand binds to the protein with a stabilizing effect. On the contrary, for CDCA a lower value of unfolding temperature was observed. Usually, the thermodynamic parameters of unfolding can be determined using CD spectroscopy when the unfolding process is reversible, but in the case of FXR-CCEE, the process is irreversible [41].

Finally, we used ITC to evaluate the thermodynamic aspects of CAF binding. As in the case of CDCA, the binding of CAF has a mainly entropic character. This hypothesis is supported by the results of the docking study, considering that the binding of cafestol to the receptor has a hydrophobic character (mainly CH-π and π-π interactions). Such interactions are weaker compared to those involved in the binding of the more hydrophilic GW4064 and are comparable to the binding of the natural ligand CDCA. Therefore, while in the case of GW4064, a considerable heat exchange can be expected when hydrophilic interactions are formed, a much lower enthalpy contribution is expected upon binding of the receptor to either cafestol or CDCA.

## 4. Materials and Methods

### 4.1. Materials

All chemical reagents used were of analytical grade and obtained commercially.

CAF and 16OMC standards were obtained from PhytoLab (Vestenbergsgreuth, Germany), and their purity was ≥99%. Chenodeoxycolich acid (CDCA), GW4064, guggulsterone (GG), DMSO and methanol (MeOH) were purchased from Sigma-Aldrich (Saint Louis, MO, USA). Stock solutions of these compounds were prepared by dissolving them in DMSO, kept at 4 °C, and diluted to the required experimental sample concentration.

### 4.2. Protein Preparation

The wild-type LBD of FXR (residues 225–472) (FXR-WT) was expressed as a recombinant protein in *Escherichia coli* cells using a plasmid kindly provided by Prof. Jinsong Liu (Guangzhou Institutes of Medicine and Health, Chinese Academy of Sciences), containing the gene sequence of the wild-type protein with a 6× histidine N-terminal tag. The C432E/C466E (CCEE) mutant (FXR-CCEE) was obtained by two sequential site direct mutagenesis reactions on the same plasmid, with the following forward and reverse primers provided by Sigma-Aldrich: ACACTTTGCCGAGCTCCTGGGTC and TGAGGATTTTCAGGCTGG for the first reaction, and CCCACTTCTCGAGGAAATCTGGGAC and GTAAACTTGTGGTCGTTTAC. Q5 High-Fidelity DNA Polymerase (New England BioLabs, Ipswich, MA, USA) was used for these reactions, following the instructions of the supplier.

Protein expression was obtained as previously reported [42], both for the wild-type protein and for the CCEE mutant. Briefly, *E. coli* BL21(DE3) cells were transformed by heat shock and grown at 25 °C in LB supplemented with kanamycin (50 μg/mL). When the optical density of the culture, measured at 600 nm, reached 1, the protein expression was started by the addition of IPTG 0.1 mM, and the incubation temperature was reduced to 16 °C. Cells were harvested 16 h after induction.

Cells were lysed in Tris 20 mM, pH 8.2, NaCl 150 mM, glycerol 10%, 1% Tween 20, 1 mM PMSF (phenylmethylsulfonyl fluoride), imidazole 10 mM, using an ultrasonic homogenizer. The protein was purified in two steps: an immobilized metal affinity chromatography (IMAC) purification and a size exclusion purification. The IMAC was conducted in batches using a Co-NTA resin (Talon Superflow, Merck, Darmstadt, Germany) and eluting the protein with Tris 50 mM, pH 8.2, NaCl 500 mM, glycerol 10%, imidazole 150 mM. Fractions containing the protein were pooled and concentrated on a 10 kDa-cutoff centrifugal concentrator. The solution was injected in a Superdex 200 10/30 Increase column, mounted on a GE Healthcare AKTA FPLC system, and the protein was eluted with Tris 20 mM, pH 8.2, NaCl 150 mM, glycerol 10%. Protein purity was assessed by SDS-PAGE with 12% acrylamide. Fractions containing the protein were pooled and stored at −80 °C until use.

### 4.3. Fluorescence Analysis

A protein aliquot was thawed in ice and dialyzed against a 50 mM phosphate buffer, pH 7.4, 500 mM NaCl, and 10% glycerol, using an 8 kDa cutoff membrane.

All steady-state fluorescence spectra were recorded at 25 °C using a CARY Eclipse (Agilent) spectrofluorometer equipped with a 0.5 cm path length quartz cuvette or a Perkin-Elmer LS-55 instrument. The excitation wavelength was set at 280 nm (λ_exc_), and emission spectra were recorded from 300 to 400 nm. For the synchronous fluorescence spectra (SFS), Δλ was set at 60 nm in order to selectively observe the changes in tryptophan emission, and the SFS were recorded from 260 to 320 nm. The slit width on the excitation and emission wavelengths were set to 5 nm for the titrations with GW4064 or GG, carried out in a 1.0 cm path length quartz cuvette, and to 10 nm for the titrations with the other ligands, obtained with a 5 mm path length quartz cuvette. Experiments with GW4064 and GG were performed with a constant FXR-CCEE concentration of 10 μM, in 500 μL of phosphate buffer; all other measurements were performed with a protein concentration of 1 μM, in 500 μL of phosphate buffer. GW4064 and GG concentrations were increased from 1 μM to 30 μM by adding aliquots of their stock solutions. Diterpene concentrations were changed in the same way from 100 nM to 40 μM. The final amount of DMSO was always lower than 10%, and it has been verified that such an amount of solvent does not affect the fluorescence of FXR-CCEE. After the addition of each ligand, the emission spectra were recorded with a delay of 3 min. All the analyses were replicated three times. Fluorescence data were analyzed using ExcelTM (Microsoft OfficeTM) and Veusz (https://veusz.github.io/). Slight inner filter effects in the titration with the diterpenes were observed due to their modest absorbance at 280 nm and were corrected according to our previous studies on the interactions of diterpenes with albumins [43]. Conversely, quenching data for the titrations with GW4064 and GG were not corrected for inner filter, as the aim was to replicate and compare the behavior of our expressed proteins with the literature data of Yang et al. [39] which are apparently not corrected for inner filter. A fluorescence lifetime of 5 ns was chosen for tryptophan in Stern–Volmer analysis of quenching by ligands [44].

### 4.4. Circular Dichroism Spectroscopy

Circular dichroism (CD) measurements were carried out using a Jasco J-715 Spectropolarimeter and an external Argolab CB5-10 thermocryostat for the control of the temperature. Experiments were performed using a 0.2 cm path-length quartz cuvette. A wavelength range of 200–240 nm was selected using a scan speed of 20 nm/min, and four scans were averaged. All the analyses were replicated twice. The raw data, expressed in mdeg values, were transformed in mean residue molar ellipticity (deg·cm^2^·dmol^−1^) using the following equation: [Θ]_λ_ = CD signal (deg)·MRW/concentration (g/L) × l × 10, where MRW is the mean residue weight (117.53) and l is the path length of the CD cell in cm.

For the circular dichroism experiments, a protein aliquot was thawed in ice and dialyzed overnight against a phosphate buffer solution containing 1.06 g/L Na_2_HPO_4_ and 0.61 g/L KH_2_PO_4_, pH adjusted to 7.35 with NaOH. The protein concentration was determined by measuring the absorbance of the solution at 280 nm and using the extinction coefficient calculated from the protein primary structure using the ProtParam web tool (18,450 M^−1^ cm^−1^) [45].

The following mother solutions of the ligand in methanol were used: 3.30 mM for cafestol, 3.25 mM for 16OMC, and 1.27 mM for CDCA. A final concentration of 5 μM of protein was used for CD measurements. CD spectra of the protein were registered immediately upon the addition of the ligand in protein/ligand ratios of 1:10, 1:50, and 1:100, respectively. Each spectrum was baseline corrected for buffer and ligand.

Temperature-induced denaturation curves of the protein were performed in the range 15 °C to 72 °C using a three-degree step and a delay of 5 min to reach the temperature before registering the spectra. The denaturation assay was performed by keeping the concentration of FXR-CCEE fixed at 5 μM in phosphate buffer for all the measurements.

Temperature-induced denaturation curves of the protein in the presence of the ligand were registered in the same conditions using FXR-CCEE and diterpenes at 5 μM and 40 μM, respectively, while FXR-CCEE and CDCA at 5 μM and 50 μM, respectively.

### 4.5. Molecular Modelling

The calculations were carried out using the Schrodinger molecular modeling suites. Optimizations of the proteins were performed in the AMBER14 force field. All the models were built starting from the crystallographic structure of FXR complex with CDCA. The geometry of the ligand-binding domain of FXR was downloaded from the Protein Data Bank, structure 6HL1 [46]. The model was thermalized with a dynamic run and finally optimized for the further docking studies.

### 4.6. Molecular Dynamics Simulations

Molecular dynamics simulations were run for 400 ns using the Gromacs suite [47], starting from the models of FXR-LBD in complex with CDCA and FXR-LBD in complex with cafestol, obtained from docking analyses. Considering that the starting model was missing six residues, not visible in the electron density of the crystallographic structure, the missing residues of the protein model have been reconstructed using the SWISS-model server by homology modeling starting from the PDB structure 6HL1 as a template [48]. The missing loop is not involved in the binding pocket. Protein and ligand complexes have been built, superimposing the reconstructed model with the docked models of FXR-LBD-cafestol and FXR-LBD-CDCA complexes.

Both complexes were prepared for MD simulation following the same protocol: Topology of the ligand was calculated using Antechamber and the general Amber force field [49]. The protein topology was obtained with the Amber ff99SB-ILDN force field instead [50,51,52]. Both FXR-LBD-ligand complexes were placed in a cubic box with explicit TIP3P water molecules and at least 10 Å of solvent on all sides of the protein. To reach physiological conditions Na^+^ and Cl^−^ ions were added neutralizing the system to a final concentration of 150 mM of salt. Systems were firstly minimized and relaxed, adapting an equilibration workflow published by Roe and Brooks for the GROMACS package [53], followed by 400 ns of NPT run at 300 K, with a time step of 2 fs using a modified Berendsen thermostat and a Parrinello-Rahman barostat.

### 4.7. Isothermal Titration Calorimetry (ITC) of FXR-CCEE

An aliquot of FXR-CCEE protein was thawed in ice and dialyzed overnight against Tris 50 mM pH 8.0, NaCl 150 mM, glycerol 5%, at 4 °C. Protein concentration was measured after the dialysis using the spectrophotometric method. ITC experiments were performed on a MicroCal ITC200 instrument (Malvern Panalytical, UK) equipped with a Hastelloy measurement cell of 200 μL volume and a titration syringe of 40 μL volume. All the measurements were performed maintaining the measurement cell at a constant temperature of 25.00 ± 0.01 °C. To perform ITC experiments, the protein was diluted to a concentration of 47 μM with the dialysis buffer, and 5% of DMSO was added to match the titrant buffer composition. GW4064, CDCA, and CAF were used as titrants in different experiments, with a concentration 20 times higher than the protein, i.e., 920 μM. Ligands were diluted from stock solutions in DMSO to match the protein buffer and avoid mismatch signals in the ITC analysis. All samples were centrifuged for 15 min at 13,000 rpm and then degassed before ITC analysis. 280 μL of protein sample was loaded with FXR solution in order to fill the calorimeter cell and the loading stem. Titrations were performed by 20 sequential additions of 2 μL of ligand solution in 4 s (except for an initial addition of 0.4 μL in 0.8 s), 60 s initial delay, and 180 s delay after each addition. A stirring speed of 750 rpm was set during the titrations in order to ensure rapid mixing of the solutions after each injection. An additional titration of the ligand into the cell containing only the buffer was also performed to evaluate the dilution heats and the buffer mismatch, which were found to be negligible. Data analysis was performed using NITPIC 1.1.5, SEDPHAT 12.1, and GUSSI 1.1.0 software packages [54,55,56].

## 5. Conclusions

The interaction of cafestol and 16OMC with the mutant type of FXR was demonstrated by means of fluorescence spectroscopy and circular dichroism.

A fluorescence quenching was observed for both cafestol and 16OMC regarding the tryptophan residues due to an interaction occurring in the close environment of the tryptophan residue W454 of the protein, located at the bottom of the binding site. The Stern–Volmer analysis shows a bimodal behavior of the quenching phenomenon, with two linear regions, one at low concentrations of ligands with a higher slope and the second at higher concentrations with a lower slope. The change of slope occurs at about 1 μM in both cases. The K_SV_ values were calculated according to the Leher equation as reported by Yang et al. in 2016 [40] and were shown to be 8.54 μM for CAF and 7.62 μM for 16OMC, values comparable with those reported by Yang for prenylflavonoids and bile acids as well. Since the change of slope occurs actually at ligand concentrations corresponding to a 1:1 molar ratio with the protein, this could be the consequence of tight binding titration of a high-affinity binding site placed close to one tryptophan side chain. Meanwhile, a second binding site would be occupied in the high concentration range, with affinities in the 10 μM range.

This hypothesis is confirmed by molecular docking, where both the terpene ligands are found to partially occupy the binding site for the natural ligand CDCA, placing the aromatic furan ring at the bottom of the site, close to Trp 454. The presence of a strong interaction between cafestol and Trp 454 was confirmed by analyzing the trajectories of the MD simulations, in which a concerted movement of the ligand and the aromatic residue was observed. This agrees with the fluorescence results, as only the environment around Trp 454 could change upon binding the terpenes. Further confirmation of the interaction was obtained by analysis of the circular dichroism of the FXR in the presence of different concentrations of ligands. A conformational change is observed after the addition of the ligands, which is more evident with CAF with respect to 16OMC and CDCA. The analysis by circular dichroism of the thermal unfolding of the protein confirmed the interaction between the two diterpenes and FXR since a slight shift to higher values was observed for the unfolding temperature.

These results provide an evidence at molecular level that confirm the hypothesis of Ricketts on the action of CAF as an agonist of FXR [21], based upon biological tests on mice and cellular models, suggesting that the competition of the terpenes with the endogenous ligand CDCA can result in the cholesterol enhancement in blood serum.

Experiments to explore in the future to further confirm the thermodynamics of the binding of cafestol to the FXR receptor include displacement isothermal titration calorimetric experiments. Moreover, the crystal structures of the complexes between the FXR domain and the two ligands CAF and 16OMC would corroborate our docking analysis.

## Figures and Tables

**Table 1:** Quenching and affinity data from Stern–Volmer analysis of fluorescence titrations suggest a static mechanism of binding, both in the low and high ligand concentration ranges.

	Low Range			High Range		
	K_SV_ (L mol^−1^)	K_q_ (10^13^ L mol^−1^ s^−1^)	K_d_ (μM)	K_SV_ (L mol^−1^)	K_q_ (10^13^ L mol^−1^ s^−1^)	K_d_ (μM)
CAF	117,050 ± 11,430	2.34 ± 0.23	8.54 ± 0.83	13,780 ± 120	0.27 ± 0.02	72.56 ± 0.05
16OMC	131,160 ± 12,280	2.62 ± 0.25	7.62 ± 0.71	24,690 ± 1520	0.49 ± 0.31	40.50 ± 2.29

**Table 2:** Unfolding temperature of FXR-CCEE with 15% methanol and in the presence of CDCA, CAF, and 16OMC at 210 nm and 225 nm, indicate an increased stability of the protein upon binding to the coffee terpenes and a destabilizing effect of the natural agonist CDCA.

λ (nm)	FXR + 15% MeOH	FXR + CDCA	FXR + CAF	FXR + 16OMC
210 nm	51.18	49.81	53.84	52.75
225 nm	52.54	51.38	55.25	54.31

**Figure A6:**
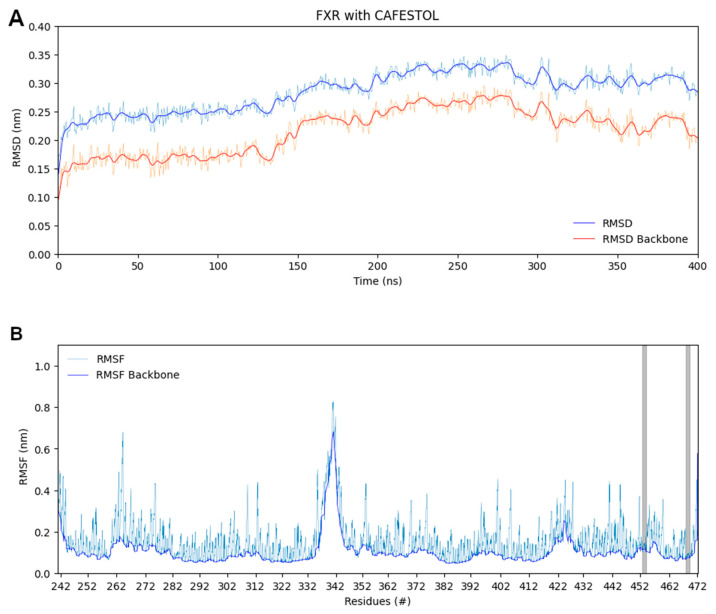
Four hundred nanosecond MD simulation of the CAF ligand bound to the LBD of FXR. (**A**) RMSD of the protein along the trajectory showing that a single conformational state is occupied by the protein. Thick lines represent the curves smoothed using a Gaussian filter at 3.0 σ. (**B**) RMSF of protein residues confirms a higher mobility of Trp 454 compared to Trp 469 (in grey). A significant movement of the loop between helices H5 and H6 can be observed between residues 340 and 344 in this plot, confirming previous results from Kumari et al. [57]. Thick lines represent the protein backbone RMSF.

**Figure A7:**
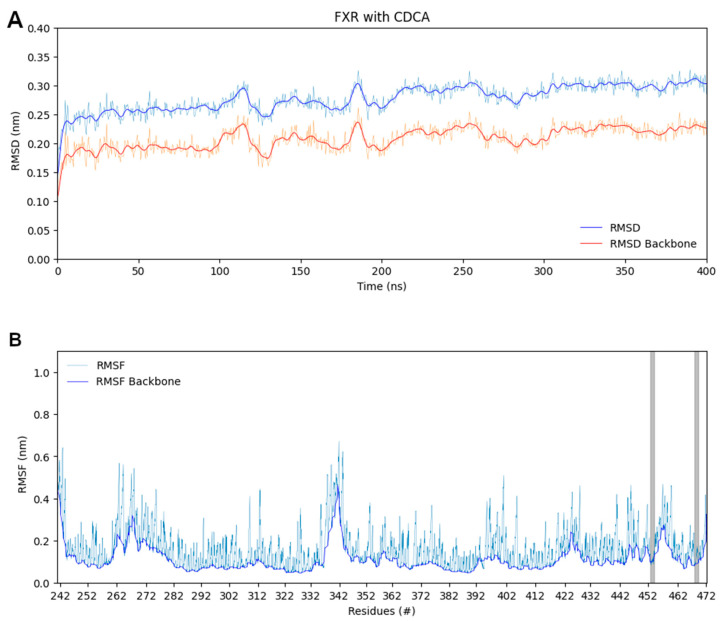
Four hundred nanosecond MD simulation of the CDCA ligand bound to the LBD of FXR. (**A**) RMSD of the protein along the trajectory showing that a single conformational state is occupied by the protein. Thick lines represent the curves smoothed using a Gaussian filter at 3.0 σ. (**B**) RMSF of protein residues confirms that Trp 454 and Trp 469 (in grey) have similar mobility. A significant movement of the loop between helices H5 and H6 can be observed between residues 340 and 344 in this plot, confirming previous results from Kumari et al. [57]. Thick lines represent the protein backbone RMSF.

**Figure A8:**
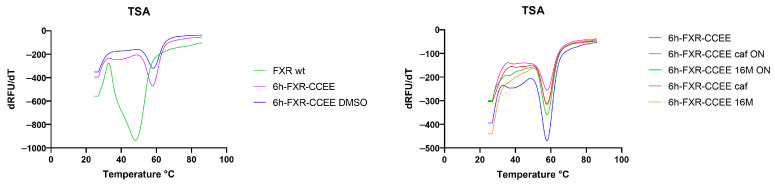
Thermal shift assay of FXR wt and FXR-CCEE with diterpenes.

## Data Availability

Data are contained within the article and Supplementary Materials.

## Supplementary Materials

The following supporting information can be downloaded at https://www.mdpi.com/article/10.3390/ijms25116096/s1.

## Appendix B

Results of the ITC measurements show that, while a significant exothermic binding is observed for GW4064 (Figure A4, Table A1), no significant heat exchange can be measured when the protein is titrated with either CAF or the natural agonist CDCA, indicating that the binding has a mainly entropic character.

**Table A1:** Thermodynamic parameters of GW406 binding to FXR, as obtained by ITC measurements.

	K_d_ (μM)	ΔH (kcal mol^−1^)	Stoichiometry	ΔG (kcal mol^−1^)	ΔS (cal mol^−1^ K^−1^)
replica 1	0.975	−6.49	0.876	−8.200	5.725
replica 2	0.883	−4.48	1.225	−8.259	12.676
mean	0.93 ± 0.07	−5 ± 1	1.0 ± 0.2	8.23 ± 0.04	9 ± 5

## Appendix D

The thermostability of both proteins (FXR and FXR-CCEE) was analyzed using a thermal shift assay with a Biorad CFX Connect Real-Time System. The assays were performed in 96 well plates in independent triplicates (Brand). Briefly, a 25 µL solution containing 2 µM protein, Tris 20 mM, pH 8.2, NaCl 150 mM, glycerol 10%, and 5× Sypro Orange protein gel staining (ThermoFisher, Waltham, MA, USA) 10 µM diterpenes underwent a denaturation cycle from 24 to 86 °C in incremental steps of 0.5 °C/min, the first derivative of the melting curve was calculated. The table shows how the mutant has a higher thermal stability than the wild-type protein and that the interaction with the diterpenes is not affecting its stability.

**Table A2:** Thermal stability of the proteins FXR wild type and FXR-CCEE and FXR-CCEE in the presence of diterpenes.

FXR wt	FXR-CCEE	FXR-CCEE + CAF	FXR-CCEE + 16OMC	FXR-CCEE + DMSO
48 °C	58 °C	58.5 °C	58.5 °C	58.5 °C
