# Supplementary material for: On the Cholesterol Raising Effect of Coffee Diterpenes Cafestol and 16-O-Methylcafestol: Interaction with Farnesoid X Receptor
Source: Int J Mol Sci. 2024 May 31;25(11):6096. doi: 10.3390/ijms25116096 (PMC11173301; doi:10.3390/ijms25116096)
Supplement: Supplementary file 1 [file ijms-25-06096-s001.zip › ijms-3017711-supplementary.pdf]

Movie S1. Trajectory of the 400 ns MD simulation of the CDCA ligand bound to the LBD of FXR. The protein is represented as a white cartoon, the CDCA ligand is represented as red spheres, and the Trp residues 454 and 469 are represented as blue and grey spheres, respectively.

Movie S2. Trajectory of the 400 ns MD simulation of the CAF ligand bound to the LBD of FXR. The protein is represented as a white cartoon, the CAF ligand is represented as red spheres, and the Trp residues 454 and 469 are represented as blue and grey spheres, respectively.
